# Myeloid-Derived Suppressor Cells: Function, Migration, and Therapeutic Opportunities in Glioblastoma

**DOI:** 10.3390/cells15121099

**Published:** 2026-06-17

**Authors:** John W. Figg, Caitland Love, Illeana West, Dan Jin, Mia Engelbart, Dorothy Ware, Rachael Bessey, Catherine T. Flores

**Affiliations:** Preston A. Wells Jr. Center for Brain Tumor Therapy, Lillian S. Wells Department of Neurosurgery, University of Florida, Gainesville, FL 32610, USA; jwfigg@ufl.edu (J.W.F.); dorothy.ware@ufl.edu (D.W.);

**Keywords:** MDSC, glioblastoma, chemokine, migration, immunotherapy

## Abstract

Myeloid-derived suppressor cells (MDSCs) are a class of immature, heterogenous, and functionally immunosuppressive myeloid progenitors that are expanded in malignant disease including glioblastoma (GBM). Extensive preclinical evaluation of GBM has revealed that MDSCs express multiple different chemokine and cytokine receptors that facilitate their entry, infiltration, expansion and immunosuppression of antitumor immunity in the tumor microenvironment. Additionally, translational investigation of approaches that target MDSCs directly or indirectly through immune remodeling has yielded promising effects that are under clinical trial investigation. Given the immunosuppressive phenotype of high-grade gliomas like GBM, the removal of MDSCs represents a clinically relevant strategy to enhance immune responses against neoplastic cells. In this review, we provide a comprehensive summary of MDSCs in GBM, emphasizing clinical observations and large-scale multi-omics studies that position MDSCs at the nexus of GBM immunosuppression. Next, we provide detailed coverage of multiple chemokines, cytokines, and growth factors that are relevant to MDSC migration, survival and expansion in GBM along with commentary on the associated receptors. Lastly, we discuss therapeutic approaches that directly target MDSCs as a strategy to improve immune responses against malignant brains and observations on the changes to MDSCs in the tumor microenvironment after immunotherapy. Our review serves as a valuable resource for the neuro-oncology research space, updating scientists and clinicians on a cell central to the biology and therapeutic targeting of GBM.

## 1. Introduction

Glioblastoma (GBM) is the most common primary malignant brain tumor diagnosed in adults and remains a major contributor to central nervous system cancer-related morbidity and mortality [1]. Standard-of-care therapy consists of maximal safe surgical resection followed by radiotherapy and temozolomide; however, nearly all tumors recur, and durable responses remain uncommon [1]. Treatment failure is driven by multiple overlapping features of GBM biology, including diffuse invasion into the surrounding brain, profound intratumoral heterogeneity, therapy-resistant cellular states, and an immunosuppressive tumor microenvironment (TME) [2]. Molecular and transcriptional profiling studies have further classified GBM into biologically distinct subtypes, including classical, mesenchymal, proneural, and neural groups, although more-recent studies suggest that these programs can coexist as dynamic cellular states within individual tumors [3,4]. These subtype or state differences are clinically relevant because they are associated with differences in oncogenic signaling and immune composition, as well as therapeutic resistance and clinical behavior [3]. Mesenchymal and recurrent GBM states are frequently associated with inflammatory and myeloid-enriched immune landscapes, suggesting that tumor-intrinsic programs may shape local immunosuppression and influence response to therapy [5,6].

Among the immune populations enriched in GBM, myeloid-derived suppressor cells (MDSCs) have emerged as central regulators of antitumor immune dysfunction [7,8,9]. MDSCs are immature myeloid cells that arise during altered or emergency myelopoiesis in settings of chronic inflammation, infection, tissue injury, and malignancy [7]. Physiologically, immature suppressive myeloid populations can restrain excessive immune activation and contribute to tissue repair; however, in cancer, these programs are pathologically co-opted to support immune evasion and tumor progression [8]. First described in mouse models of kidney allograft tolerance, MDSCs characteristically expressed CD11b+ (Itgam), Ly6C (Ly6c1/2), and Ly6G (Ly6g) and functionally suppressed T cell activation through nitric oxide synthase (NOS)- and arginase-1-dependent mechanisms [10]. In GBM, both human and murine studies have identified multiple MDSC populations [9,11,12]. In human GBM, early MDSCs (e-MDSCs) and monocytic MDSCs (M-MDSCs) have been annotated based on stem cell-associated programs and CD14 expression, respectively [9]. Similarly, murine GBM models contain M-MDSCs, defined by Ly6ChiLy6G− status, as well as granulocytic or polymorphonuclear MDSCs (PMN-MDSCs), defined by Ly6CloLy6G+ status [13,14,15,16].

In cancer, MDSCs expand in the bone marrow, peripheral blood, and TME, where they reprogram local and systemic immunity toward a suppressive state that limits T cell activation and antitumor immune function [11,12,17,18,19,20]. In GBM, MDSC enrichment has been associated with adverse clinical outcomes like therapeutic resistance and disease recurrence. Iatrogenic elevation of MDSCs has been observed after temozolomide or radiotherapy in subsets of patients who develop profound lymphopenia, a state correlated with poorer treatment response and shorter two-year overall survival (OS) [21,22,23]. In recurrent GBM, MDSC enrichment is associated with worse OS, and circulating M-MDSC levels have been correlated with a poorer response to boron neutron capture therapy [11,24]. Biological sex further modifies MDSC biology in GBM. Male GBM samples are enriched for M-MDSCs, whereas PMN-MDSC signatures are enriched in female GBM samples, with higher enrichment associated with poorer prognosis [25]. Transfer of male M-MDSCs in murine GBM models enhanced tumor progression, an effect blocked by DPP4 inhibition through reduced pERK signaling and decreased MDSC migration [26]. Together, these findings indicate that MDSC abundance, along with subset composition and their suppressive function, is a clinically relevant feature of GBM immunobiology.

Mechanistically, MDSCs suppress antitumor immunity through multiple complementary pathways. Production of reactive nitrogen and oxygen species promotes T cell dysfunction and apoptosis, while additional mechanisms include arginine depletion, cysteine/cystine sequestration, nitration of T cell co-stimulatory molecules, and interference with antigen-specific T cell responses through MHC class I-dependent interactions [10,27,28,29,30,31]. Beyond these suppressive effector mechanisms, extensive investigation has identified a broad network of chemokines, cytokines, interferons, colony-stimulating factors, and growth factors that regulate MDSC recruitment, expansion, survival, and functional programming in GBM. Because many of these pathways are redundant and context-dependent, understanding how they converge on MDSC biology is critical for designing therapeutic strategies that remodel the GBM immune microenvironment without unintentionally enhancing tumor-promoting inflammation or impairing antitumor immune responses.

**Figure 1:**
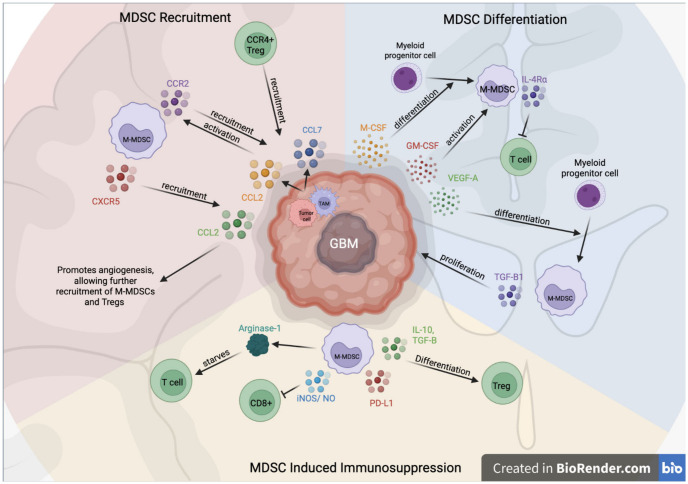
Myeloid-derived suppressor cell-associated chemotactic milieu in glioblastoma. Schematic depiction of secretory molecules and their impact on myeloid-derived suppressor cell (MDSC) recruitment, differentiation and immunosuppression. MDSC recruitment is stimulated by CCL2 and CCL7 chemokines through CCR2 signaling. MDSC differentiation is stimulated by colony-stimulating factors and growth factors derived from the surrounding microenvironment. MDSC-induced immunosuppression is mediated by arginase-1 activity in addition to growth factors like IL-10 and TGF-β.

In this review, we summarize the role of MDSCs in GBM with an emphasis on the molecular pathways that regulate their migration, expansion, and suppressive function (Figure 1). We first provide an overview of chemokine, cytokine, interferon, colony-stimulating factor, and growth factor axes implicated in MDSC biology, highlighting receptor–ligand interactions, cellular mechanisms, clinical correlates, and evidence from GBM models where available. We then discuss therapeutic strategies that directly target MDSCs or indirectly remodel MDSC-associated immunosuppression, including chemokine receptor blockade, differentiation therapy, phosphodiesterase inhibition, growth factor pathway targeting, and immunotherapy-based approaches. Finally, we synthesize these findings into a framework for understanding MDSCs as both biomarkers of GBM immune dysfunction and candidate therapeutic targets for improving responses to current and emerging therapies.

## 2. C-C Chemokine Receptor and Ligand Axes

The CCL2-CCR2 axis has emerged as a dominant stimulator of MDSC migration in gliomas (Figure 1). Multiple ligands bind to CCR2 including CCL2, CCL7, CCL8, CCL12, CCL13, and CCL16 [32,33,34,35,36]. In human and murine GBM, both neoplastic cells and TAMs contribute to the production of CCL2 and other ligands to CCR2 like CCL7 and CCL12 [36,37,38,39,40]. In preclinical animal models, NOTCH-activated TAMs amplify CCL2 secretion, thereby stimulating MDSC migration in animal models of GBM, and analogous CCL2 induction was also observed in serum in human rGBM samples [41]. The source of CCR2 ligands is particularly relevant to glioma immunosuppression given that upwards of 50% or more of the GBM tumor mass consists of tumor-associated macrophages (TAMs), thereby facilitating immune escape and angiogenesis [42].

Glioma-derived factors like CCL20 and osteoprotegerin induce transcriptional activation of CCL2 in TAMs and surrounding tissue, creating an environment that is permissive of MDSC migration [36,43]. High levels of CCL2 in human GBM samples correlated with poorer survival in patients [19,36]. Other solid malignancies like hepatocellular carcinoma express CCL2, which promotes MDSC and TAM recruitment through CCR2, facilitating T cell suppression and tumor progression [44]. In colorectal cancer models, tumors express lactate receptor HCAR1, a GPCR that upregulates CCL2 expression, which induces migration of CCR2^+^ polymorphonuclear myeloid-derived suppressor cells (PMN-MDSCs) [39].

The enrichment of CCR2 ligands across various solid tumor subtypes prompted investigation into ligand neutralization as a strategy to subvert MDSC infiltration and recruitment (Table 1). In vitro studies of GL261 cells demonstrate that CCL2 depletion suppresses migration of MDSCs and TAMs toward tumor-conditioned medium [45]. In transgenic mouse models, neutralization of CCL2 with genetically engineered *Ccl2*^−/−^ mouse models demonstrate significant reductions in intratumoral MDSC populations in models of metastatic lung cancer, high-grade glioma and colorectal cancer [35,45,46,47,48]. Furthermore, in C57BL/6 murine models bearing intracranial GL261 glioma, CCL2 blockade was introduced via monoclonal antibody treatment. This prolonged mouse survival and decreased TAM/M-MDSC populations in the TME [48]. Small-molecule antagonists against CCR2 have demonstrated significant therapeutic benefits as monotherapy in preclinical models of GBM and enhance responses to immune checkpoint blockade (ICB) in models that are resistant to ICB monotherapy [48]. Combinatorial approaches leveraging CCR2 targeting with ICB reshaped the TME phenotype with associative reductions in MDSCs and enrichment of activated TILs [48].

Other CC chemokine family members like CCL5 (or RANTES) promote migration and infiltration of MDSCs and are associated with a poorer prognosis in cancer [49,50,51]. Targeting CCR5, the receptor for CCL5, along with CCR2 co-targeting resulted in significant depletions of M-MDSCs, ICB responsiveness and increased OS [52]. Like CCL5, CCL20 promoted M-MDSC expansion, but in vivo targeting of this chemokine abrogated this effect in addition to reducing tumor volume and extending OS against GL261 GBM [53].

## 3. CXCL-X-Mediated Recruitment

Paralleling M-MDSC recruitment with CCL2, CXCL1/2 ligands stimulate PMN-MDSC migration in models of gastric cancer, bladder cancer, cholangiocarcinoma, GBM, and other malignancies [54,55,56,57,58,59,60,61]. CXCL1 chemokine secretion in murine GBM was linked with lipid metabolism enzymes such as acetyl-CoA acetyltransferase (ACAT) where loss of ACAT promoted MDSC infiltration due to macrophage-specific CXCL1 [58]. This is not unexpected given that other metabolic pathways such as IDO and tryptophan metabolism have been shown to upregulate complement factors to promote infiltration of T-regulatory cells and MDSCs in GBM [59]. Resistance to immunotherapy in GEMM models of EGFRvIII identified significant correlations with CXCL1/2/3 expression and CXCR2^+^ PMN-MDSC enrichment in secondary lymphoid organs [60]. This association is consistent with other cancer subtypes where markedly increased expression of CXCL2 was associated with shorter OS in patients with bladder or ovarian cancer [56,57,61]. As previously described in the CCL2-CCR2 axis, CCL20 stimulates transcriptional activation of CXCL2, along with other transcriptional programs driven by S100A9, and galectin-1 [53]. Other CXCL members such as CXCL12 stimulate bone marrow mobilization of CXCR4^+^ MDSCs and promote immunosuppressive programs in GBM [62,63,64,65]. Blockade of the CXCL12-CXCR4 axis polarized macrophages from M2 phenotypes to M1 phenotypes, reduced MDSC infiltration, and enhanced cytotoxic T cell activity [62,63,64,65]. Nanoparticle-based platforms against the CXCL12-CXCR4 axis similarly reduce immunosuppressive myeloid cell populations and promote cytotoxic lymphocyte responses with ICB in orthotopic GBM murine models [65]. CXCR4-specific targeting combined with ICB also enhanced pro-inflammatory signatures and immune memory, leading to significantly increased OS in murine GBM relative to monotherapy alone [64,65]. Another family member, CXCL8, promotes tumor angiogenesis, epithelial-to-mesenchymal transition, and recruitment of MDSCs to promote immune escape [66,67,68]. Increased levels of CXCL8 in patients were associated with worse clinical outcomes and poorer OS after immunotherapy treatment [69]. Neutralization of CXCL8 augmented efficacy of ICIs, decreased MDSC accumulation, increased polyfunctional TILs, and delayed tumor resistance [69].

## 4. Colony-Stimulating Factor Recruitment

Whereas CCL2 and CXCL1/2 primarily induce MDSC recruitment, the colony-stimulating factor family members M-CSF and GM-CSF drive expansion and functional programming of MDSC populations in cancer (Table 1). M-CSF promotes T cell suppression through binding to CSF1R, leading to induction of M-MDSC differentiation [70]. Some evidence suggests that GM-CSF may induce MDSC migration, but largely this factor induced MDSC accumulation from bone marrow sources [70,71,72,73,74,75,76]. GM-CSF silencing in gliomas polarizes monocytes towards MDSC functionality through induction of IFNγR1/2 expression and nuclear translocation of IRF-1 [73,74,75]. Other transcriptional changes include expression of IL-4Rα on infiltrating MDSCs, which promotes IL-13-mediated activation of arginase-1 and subsequent T cell suppression [76]. Associated receptors for colony-stimulating factors are also implicated in GBM. CSF1R^+^ cell enrichment in the GBM TME is associated with a worse prognosis in various cancer subtypes [95]. Genetic knockdown of CSF1R in murine glioma models significantly reduced myeloid cells and TAMs from brain tumor tissue and secondary lymphoid organs, positioning this receptor as a critical regulator of the TME phenotype [77]. While colony-stimulating factors and their receptors lie at the nexus of MDSC infiltration in GBM, another class of secreted factors, the macrophage migration inhibitory factor family, has been implicated in MDSC migration in GBM. Cell sourcing of MIF in the glioma TME was derived from cancer stem cells, promoting arginase-1 activity through CXCR2 in TME-based MDSCs [78]. Inhibiting MIF with ibudilast against CD47^+^ M-MDSCs in murine glioma models significantly decreased MDSC infiltration and promoted CD8 T cell cytotoxicity in the TME, demonstrating the role of MIF in MDSC migration and immunosuppression [78].

## 5. Interleukin/Interferon Recruitment

Complementing CSF-driven regulation of MDSC expansion, interferon/interleukin families further shape MDSC differentiation and immunosuppressive function. Type I interferons decrease differentiation and maturation of MDSCs, thereby abrogating their immunosuppressive capacity against cytotoxic T cells [5,79]. However, MDSCs counter these actions by reducing responsiveness of splenocytes to interferons through nitration of STAT1 [80,81]. Unlike interferons, interleukin family members IL-6 and IL-10 induce MDSC migration and enhance their suppressive functions. IL-6-mediated signaling through the Hedgehog pathway and activates STAT3 to stimulate MDSC expansion and expression of immune checkpoint molecules [82,83]. In addition to its stimulatory role, IL-6 promotes the recruitment of MDSCs to neoplastic tissue and is highly correlated with MDSC enrichment and poorer patient outcomes [84,85,86,87]. Interestingly, radiograph modeling of human GBM suggests that T-regulatory cells occupy a central interface between IL-6 signaling and MDSC [88]. IL-10 is canonically immunosuppressive through its role in activating T-regulatory cells and suppressing T cell proliferation [89]. When MDSCs are treated with exogenous IL-10, their suppressive activity is significantly increased [90]. In states of chronic inflammation, MDSCs secrete IL-10, which dampens antitumor immunity through reductions in CD62L expression on T cells [91,92]. While anti-inflammatory signaling impacts MDSC recruitment and expansion in GBM, pro-inflammatory signaling through the NLRP3 inflammasome and downstream cytokines, IL-1β and IL-18, induced recruitment of PMN-MDSCs in murine models of GBM (subcutaneous and intracranial compartments) [93]. This is consistent with other observations that MDSC/macrophage enrichments and pro-inflammatory IFNγ signaling both associate with slow-cycling GBM cell markers like membrane-associated protein 17 (*MAP17*) [94]. Pro-inflammatory signaling through IFNγ also primes glioma-derived extracellular vesicles to promote MDSC differentiation and suppression of T cell activation [95]. Despite observed associations between pro-inflammatory signaling and MDSC enrichment, therapeutic induction using retrovirus approaches to promote interferon regulatory factor 8 enhances TIL infiltration into murine GBM while lowering MDSC Arg-1 and IDO1 activity [96].

## 6. Growth Factor Recruitment

Two other cytokines that have significant relevance to HGGs and MDSCs are VEGF family members, and TGF-β. VEGF-A is significantly increased in GBM tissues and associated with increased MDSC differentiation [97]. Other members like VEGF-C induced expression of chemokines from lymphatic endothelial cells thereby promoting CXCR2-dependent MDSC migration in a dose-dependent manner [98]. Increased plasma VEGF-C was associated with increased circulating immature myeloid cells in cancer patients [98]. Signaling from VEGF pathways promote TGF-β secretion from MDSCs, but conflicting evidence suggests that TGF-β may have opposing functions on immunity [99]. In colorectal cancer models, TGF-β is highly prevalent and promotes myeloid immunosuppression while reducing HLA-DR expression [100]. Similar impacts were observed in the bone marrow where treatment with TGF-β polarized hematopoiesis toward immunosuppressive cell types and inhibited proliferation of stimulated T cells [101]. TGF-β1/2 is also associated with FAT atypical cadherin 1 (FAT1) expression in GBM [102]. Knockdown approaches with siRNA against FAT1 significantly decreased TGF-β1/2, although this effect promoted migration of monocytes [102]. Other regulators such as exosomal long coding RNA AGAP2-AS1 bind to miR-486-3p in MDSCs to upregulate TGF-β1/2 and enhance GBM cell migration and proliferation in vitro [103]. However, TGF-β1 has been shown to exert immune stimulatory functions on MDSCs such as antigen presentation on MHC class I molecules and enhanced T cell proliferation, rendering increased tumor cell killing [103]. CAR T cell platforms have leveraged the bivalent nature of TGF-β to mount antitumor immunity and prolonged survival in models of glioma [104]. Other family members of the tumor growth factor family like growth differentiation factor 6 promote recruitment of MDSCs in solid tumors but not in GBM [105].

## 7. Therapeutic Strategies and MDSCs

Accordingly, the link between MDSC burden and patient outcomes has prompted investigation into strategies aimed at depleting or functionally reprogramming these cells (Table 2). MDSC depletion is associated with restoration of APC-mediated activation of T cell responses, Th1-dependent antibody production, maturation of DCs, and tumor regression in animal models of cancer [12,106,107]. Targeting of MDSCs using all-trans retinoic acid achieved significant MDSC depletion by inducing their differentiation into mature myeloid effectors [108,109,110]. Other organic compounds such as triterpenoid C-28 methyl ester of 2-cyano-3,12-dioxooleana-1,9, -dien-28-oic acid abrogate MDSC ROS production, thus decreasing their immunosuppressive capacity [111]. Additionally, phosphodiesterase inhibitors like tadalafil and ibudilast target PDE-5, abrogating MDSC immunosuppression and reducing circulating MDSC ratios, but did not change OS/PFS [112]. Like the rationale for targeting PDEs, MDSC expansion and functionality is dependent on tyrosine kinase signaling, prompting evaluation of tyrosine kinase inhibitors on MDSCs in GBM. Tyrosine kinase inhibitors such as Sunitinib preclinically decreased MDSC infiltration in murine GBM and blood while promoting infiltrating of T cells in the TME [113]. Direct MDSC depletion using GR-1 depleting antibodies also enhanced response to immune stimulatory gene therapy, corresponding with increased median survival in preclinical murine GBM models [114]. Indirect modulation of MDSCs in GBM has also been observed after chemotherapy or immunotherapy. With respect to chemotherapy approaches, neoadjuvant low dose capecitabine reduced circulating MDSCs and promoted the infiltration of immune cells with cytotoxic phenotypes in glioma tissue prior to surgery in patients with recurrent GBM [115]. ICI treatment significantly decreased MDSCs and T-regulatory cells in models of melanoma, while peptide vaccination similarly decreased MDSC abundance [116,117]. Antagonism of chemokine receptors as previously described also synergizes with ICIs [48]. Pharmacologic targeting of inflammatory signaling provides an additional complementary strategy, as COX-2 inhibition reduces prostaglandin-mediated MDSC recruitment and suppressive function [118]. Other targeted approaches against inflammatory cascades through NOTCH signaling reversed MDSC immunosuppression following oncolytic viral therapy in murine gliomas [40]. Vascular strategies against VEGF produced limited responses, but triple therapy with anti-VEGF and blockade against Ang-2 and PD1 led to significant increases in OS against murine GBM [119].

Cellular immunotherapies also exert potent changes on MDSC populations in solid tumors through direct targeting or displacement of the tumor microenvironment. IL-13Rα2 CAR T cells fused to IL-15 led to a significant reduction in MDSCs and decreased secretion of immunosuppressive molecules [120]. CAR functionality was metabolically enhanced with metformin and mTOR to promote displacement of MDSCs [121]. CAR-based therapies have also been augmented using chemokine-loaded oncolytic viruses to stimulate CAR T cell infiltration to the GBM microenvironment [122]. Using a construct to express CXCL11, CAR T cells readily infiltrated the TME and displaced immunosuppressive MDSCs [122]. Polyclonal adoptive cellular therapy platforms and stem cell therapies such as HSC combined with ICI result in displacement of endogenous MDSCs from the GBM microenvironment [123,124]. Recent work evaluating adoptive cellular therapy in murine GBM identified significant reductions in TAM-derived CCL12, which was shown to promote MDSC migration in vitro [19]. Gene signatures associated with MDSCs were also significantly decreased after adoptive cellular therapy, shifting the TME towards more-pro-inflammatory states [19]. Similar effects with peptide therapy using tumor antigen fibrinogen-like protein 2 also reduced immune inhibitory cell populations like microglia, T-regulatory cells, and MDSCs in murine glioma while promoting increased immune memory responses, TIL activation, and loss of immunosuppressive cytokines [117].

## 8. Conclusions and Discussion

Chemoattractant signaling axes serve a vital role in the migration, expansion, and survival of MDSCs in high-grade gliomas. While MDSC expansion is observed in both the TME and blood compartments in patients diagnosed with GBM, the impact of local and systemic chemoattractant mechanisms on MDSC recruitment is less understood. Understanding of each separate mechanism is limited given that expression of the chemokine/cytokine families mentioned herein is ubiquitously found not only in the TME but also in lymph channels, cerebrospinal fluid, and serum [13,14,15,125]. Additionally, several studies that evaluated MDSC targeting utilized systemically administered neutralization antibodies to evaluate either therapeutic responses or effects on MDSCs as opposed to localized targeting within the TME [40,114]. A systemic approach against MDSCs in GBM may be efficacious given that generalized depletion of MDSCs enhanced immunotherapy responses following myeloablative chemotherapy in melanoma models [126,127]. However, GBM remains confined because of the blood–brain barrier and harbors a significantly more immunosuppressive microenvironment, supporting future investigation aimed at localized MDSC depletion in models of GBM [128].

While the impact of serum factors on MDSC recruitment remains unclear, other studies have evaluated signaling factors in the local bone marrow compartment since bone marrow-derived MDSC expansion and proliferation have been observed in patients with GBM [13,14,15]. Bone sequestration is observed across various cancer subtypes and immune subtypes including T cells in patients with GBM [129]. However, the specific factor impacting MDSCs in bone marrow also remains elusive. Recently, cholinergic and sympathetic neural signaling from the autonomic nervous system stimulated hematopoietic stem cell quiescence and expression of CXLC12 [130]. Sympathetic innervation through α-adrenergic signaling in lymphoid tissue was also shown to disrupt the accumulation of MDSCs in models of colorectal cancer [131]. Given the impact of the autonomic nervous system on blood physiology, future investigations may consider not only locally derived factors in the glioma microenvironment, but also local factors found in bone marrow that may drive enrichment and differentiation of MDSCs from hematopoietic stem cell progenitors.

Although myeloid cells such as MDSCs are highly abundant in GBM tumor tissue, immunosuppression in the GBM microenvironment is multifactorial, with contributions from regulatory lymphocytes, disease-associated microglia, and non-immune cells [132]. Additionally, conventional treatment modalities in neuro-oncology such as radiotherapy and systemic chemotherapy may worsen MDSC expansion and immunosuppression [27,126,127]. The removal or targeting of MDSCs may enhance antitumor responses, but other immunosuppressive components such as T-regulatory cells or TAMs may dampen this effect given that they secrete molecules to promote MDSC expansion, survival and persistence [14,59,117]. T-regulatory cells secrete anti-inflammatory molecules that not only promote MDSC recruitment but also inhibit IL-2 and IFN-γ release from TILs [133]. Despite complex mechanisms of immunosuppression in GBM, consolidated strategies against immune redundancies found among immunosuppressive cell types could synergize to remove microenvironment immunosuppression. Both TAMs and MDSCs in GBM express CCR2 and CSF1R, and so chemokine receptor targeting could alleviate two immunosuppressive sources, but this should be carefully considered to avoid receptors shared with TILs and other immune cells that mediate antitumor responses [35,70,108]. Leveraging approaches against shared immunosuppressive receptors is promising, but, mechanistically, it is less clear whether MDSC removal and TAM removal are mutually exclusive to driving immunophenotypic shifts in the GBM microenvironment [35,70,108,132,134].

Direct targeting of MDSCs achieved improvements in survival and prognostically significant microenvironment changes in multiple cancer models, prompting evaluation of clinical approaches. Preclinically, all-trans retinoic acid (ATRA) promoted MDSC differentiation into terminally differentiated immune cells, lessening their immunosuppressive impact [108]. ATRA was well tolerated by patients diagnosed with metastatic melanoma who also received pembrolizumab, and combined treatment was significantly associated with reductions in peripheral blood MDSCs [110]. While its effect remains inconclusive in GBM, ATRA is being evaluated with immune checkpoint blockade in recurrent IDH-mutant glioma in a trial that is open to recruitment (NCT05345002). Like ATRA, phosphodiesterase inhibitors have shown therapeutic efficacy in preclinical cancer models and significantly reduced MDSCs [135]. Clinically, tadalafil was well tolerated by patients diagnosed with GBM and circulating PMN- and M-MDSCs were significantly decreased relative to the control arm of this study (NCT04757662) [112]. Despite recapitulation of preclinical alterations to MDSC levels, it remains unclear whether direct MDSC targeting could improve survival outcomes in patients with GBM. Additionally, MDSC precision medicine may become superfluous given that various immunotherapy approaches are associated with MDSC depletion and T cell activation, lessening the need to directly inhibit MDSCs [122,136,137]. Considering the favorable safety profile of MDSC-targeting strategies, future clinical trials should investigate these approaches as treatment modifiers of immunotherapy against CNS malignancies.

## Figures and Tables

**Table 1:** Major mediator axes implicated in MDSC biology in GBM. Summary of chemokine, cytokine, interferon, colony-stimulating factor, and growth factor pathways reported to regulate MDSC recruitment, expansion, survival, or suppressive function in GBM. Where available, the table highlights the dominant MDSC population affected, supporting GBM evidence, and therapeutic relevance.

Mediator Axis	MDSC Population and Effect	GBM Evidence	Therapeutic Implication
CCL2/CCL7/CCL12-CCR2	Recruits M-MDSCs and CCR2+-suppressive myeloid cells	Glioma-derived CCL2/CCL7 and TAM-derived CCL12 promote MDSC migration in human and murine GBM [32,33,34,35,36,37,38,39,40,41,42,43,44,45,46,47,48]	CCL2 neutralization or CCR2 blockade reduces MDSC/TAM infiltration and improves survival or ICB response in preclinical glioma [46,47,48]
CCL5-CCR5/CCR2-CCR5	Supports M-MDSC infiltration and suppressive myeloid accumulation	CCR5-associated signaling is linked to MDSC recruitment in cancer and glioma-associated myeloid suppression [49,50,51,52]	Dual CCR2/CCR5 inhibition reduces M-MDSCs and enhances anti-PD-1 efficacy in glioma [52]
CCL20-associated signaling	Promotes M-MDSC expansion and recruitment	CCL20 induces suppressive myeloid programs and contributes to GL261 tumor progression [53]	CCL20/KITENIN-axis targeting reduces MDSCs, tumor burden, and improves survival in preclinical GBM [53]
CXCL1/2/3-CXCR2	Recruits PMN-MDSCs and contributes to ICB resistance	CXCL1/2/3 expression associates with CXCR2+ PMN-MDSC enrichment and immunotherapy resistance in GBM models [54,55,56,57,58,59,60,61]	CXCR2-axis targeting may reduce PMN-MDSC accumulation and improve immunotherapy response [54,55,56,57,58,59,60,61]
CXCL12–CXCR4	Mobilizes CXCR4+ MDSCs/myeloid cells and promotes suppressive infiltration	CXCL12/CXCR4 signaling contributes to MDSC recruitment and immunosuppressive myeloid polarization in orthotopic GBM models [62]	CXCR4 blockade, especially with ICB, reduces suppressive myeloid infiltration and improves survival in preclinical GBM [62,63,64,65]
CXCL8/IL-8—CXCR1/2	Promotes MDSC recruitment, angiogenesis, and ICB resistance	IL-8/CXCR1/2 signaling supports glioma immune suppression and is associated with poor outcomes in immunotherapy-treated cancer cohorts [66,67,68,69]	IL-8 neutralization decreases MDSC accumulation and improves ICB efficacy in glioma models [66,67,68,69]
M-CSF—CSF1R/GM-CSF	Drives M-MDSC differentiation, expansion, and suppressive programming	CSF1R ligands and GM-CSF promote suppressive glioma-infiltrating myeloid phenotypes, including IL-4Rα/Arg1-associated programs [70,71,72,73,74,75,76,77]	CSF1R or CSF-associated pathway targeting may reduce suppressive myeloid function, although TAM/MDSC overlap requires careful interpretation [70,71,72,73,74,75,76,77]
MIF-associated signaling	Enhances Arg1 activity and MDSC suppressive function	GBM cancer stem cell-derived MIF promotes MDSC immunosuppression; MIF receptor profiles differ across GBM MDSC subsets [78]	Ibudilast/MIF targeting reduces MDSC infiltration and enhances CD8+ T cell activity in murine glioma [78]
IL-6/IL-10/STAT3-associated signaling	Promotes MDSC expansion and suppressive activity	IL-6 and IL-10 enhance MDSC accumulation, checkpoint expression, and immunosuppressive function in GBM and other cancers [79,80,81,82,83,84,85,86,87,88,89,90,91,92]	IL-6/STAT3- or IL-10-associated pathways may be useful combination targets to reduce suppressive myeloid programming [79,80,81,82,83,84,85,86,87,88,89,90,91,92]
IL-1β/IL-18/NLRP3 and IFN-associated signaling	Regulates inflammatory recruitment and functional polarization of MDSCs	NLRP3-associated cytokines recruit PMN-MDSCs, while IFN-related pathways can either promote suppressive programming or enhance antitumor immunity depending on context [93,94,95,96]	Inflammasome or IFN-pathway modulation may reshape MDSC function but requires context-specific targeting [93,94,95,96]
VEGF-A/VEGF-C and TGF-β	Promotes immature myeloid differentiation, recruitment, and context-dependent suppressive function	VEGF and TGF-β signaling are associated with MDSC differentiation, myeloid recruitment, and immune suppression in GBM and cancer models [97,98,99,100,101,102,103,104,105]	Anti-VEGF- or TGF-β-directed combinations may reduce suppressive myeloid signaling, though effects are context-dependent [97,98,99,100,101,102,103,104,105]

**Table 2:** Therapeutic approaches targeting MDSCs or MDSC-associated pathways in GBM.

Strategy	Example Agent/Approach	MDSC-Directed Mechanism	Evidence Level	Reported Effect	Limitations
Depletion	Anti-Gr1 antibodies [12,106,107,114]	Reduces MDSC abundance	Preclinical GBM	Improved immunotherapy response	Mouse-specific marker
Differentiation	ATRA [108,109,110]	Promotes maturation of immature myeloid cells	Cancer clinical/preclinical	Reduced circulating MDSCs	GBM efficacy unclear
PDE inhibition	Tadalafil, ibudilast [112]	Reduces suppressive function/Arg1-NOS pathways	GBM clinical/preclinical	Reduced MDSCs; unclear survival benefit	Needs combination therapy
Chemokine blockade	CCR2, CCR5, CXCR2, CXCR4 inhibitors [19,32,35,36,37,48,49,62]	Reduces recruitment	Preclinical GBM	Improved ICB response/survival	Redundant pathways
CSF1R targeting	CSF1R inhibitors [35]	Reprograms/depletes suppressive myeloid cells	Preclinical GBM	Reduced myeloid suppression	TAM/MDSC overlap
VEGF-axis targeting	Bevacizumab/anti-VEGF combinations [119]	Reduces vascular and myeloid recruitment signals	GBM clinical/preclinical	Limited monotherapy benefit; combination potential	Adaptive resistance
Cellular immunotherapy	CAR T, ACT, vaccines [19,120]	Indirect displacement/reprogramming of MDSCs	Preclinical/early clinical	Reduced MDSC signatures in some models	Mechanism is often indirect
Inflammatory pathway modulation [40,56,118]	COX-2, NOTCH, MIF blockade	Reduces suppressive recruitment/function	Preclinical GBM	Improved immune activation	Context-dependent effects

## Data Availability

No new data were created or analyzed in this study.
